# Enzyme-Loaded Liposomal Edible Hydrogel Films to Enhance Lactase Activity in Perline Mozzarella

**DOI:** 10.3390/gels12040343

**Published:** 2026-04-20

**Authors:** Esin Yilmaz, Ayse Avci, Elif Sezer, Muhammad Sohail Arshad, Zeeshan Ahmad, Israfil Kucuk

**Affiliations:** 1 Institute of Nanotechnology, Gebze Technical University, Kocaeli 41400, Turkey; esinyilmaz@gtu.edu.tr; 2 Department of Food Engineering, Faculty of Engineering, Sakarya University, Sakarya 54050, Turkey; aysea@sakarya.edu.tr (A.A.); elifsezer@sakarya.edu.tr (E.S.); 3 Department of Pharmacy, Bahauddin Zakariya University, Multan 60800, Pakistan; sohailarshad@bzu.edu.pk; 4 Division of Pharmacy and Optometry, School of Health Sciences, University of Manchester, Manchester M13 9PL, UK; zeeshan.ahmad@manchester.ac.uk

**Keywords:** polyelectrolyte polymers, edible hydrogel films, lactase enzyme, shelf life, enzyme encapsulation, cheese product

## Abstract

Lactase enzyme-based products experience challenges including residual lactose that result in lactose intolerance. The purpose of this study was to develop polyelectrolyte polysaccharide-enriched lactase-encapsulated liposomal hydrogel films as an edible coating of Perline Mozzarella cheese that delivers enzymes along with the product on the side of absorption in the small intestine. Coatings were investigated for shelf-life enhancement and in vitro enzyme release behaviour. Two different polymeric hydrogel film formulations were evaluated: lactase-encapsulated liposome-enriched chitosan (PCLLa) and lactase-encapsulated liposome-enriched polyelectrolyte chitosan and sodium alginate (CLLA). Lactase-encapsulated liposomes (mean particle size: 176 nm) were produced using 20% *v*/*v* lactase enzyme and 8% *w*/*v* lecithin using probe sonication. The edible hydrogel film coatings were applied on Perline Mozzarella cheese using the standard dip-coating method. Shelf-life characteristics of all samples were evaluated using pH, colour change, dry matter determination, microbial evaluation, and sensory analysis. CLLA coatings increased shelf life up to 60 days, displaying a pH of 5.48, continued normal colour, enhanced humidity balance, minimal bacterial growth, and the highest scores for sensory values when compared to both PCLLa (coatings) and the bare cheese substrate (control) samples. Furthermore, CLLA coatings provided greater stability for liposomes within the polyelectrolyte polymeric edible hydrogel film structure. Hence, the combination of liposomes with polyelectrolyte edible hydrogel films provides a novel strategy to enhance lactase enzyme encapsulation (for intolerance), stability, and delivering ability to the small intestine as well as improving the shelf life of coated cheese products.

## 1. Introduction

Lactose intolerance is a gastrointestinal disorder affecting 65–75% of the global population [1], resulting from insufficient lactase (β-galactosidase) enzymes in the small intestine. This enzyme hydrolyses the main carbohydrate in dairy products, lactose, to form glucose and galactose molecules. A deficiency of the lactase enzyme in the small intestine results in undigested lactose. In this situation, undigested lactose reaches the colon, where it is fermented by intestinal microbiota, leading to symptoms such as bloating, abdominal pain, and diarrhoea [2]. There are two types of products on the market for lactose-intolerant individuals: (1) lactose-free/lactose-reduced dairy products and (2) pills or drops including lactase enzyme. Although lactose-reduced or lactose-free dairy products are commercially available, and they are produced by integrating lactase enzyme into milk at the early stages of production or lactose is separated by applying ultrafiltration and chromatographic separation, these approaches alter the sensory (browning, caramel-like taste, or high sugary taste) [3] and nutritional properties (loss of calcium) of the product [4]. In addition, lactose-intolerant people generally do not prefer taking pills or drops as the daily intake ratio differs according to the amount of dairy products consumed. Moreover, taking pills or drops regularly affects the psychology of consumers negatively, as they feel like patients and that they are addicted to pills. Furthermore, these people face chronic calcium malabsorption because of reduced bone mineral density and accelerated osteopenia at an earlier physiological age [5].

In recent years, edible hydrogel film and coating formation on food products have attracted attention as an innovative way to improve food functionality, quality, and shelf life [6,7]. These edible hydrogel film and coating structures can act as protective barriers against moisture, oxygen, and microbial contamination while serving as carriers for functional compounds, including enzymes, probiotics, and other bioactive molecules [8]. Edible hydrogel films and coatings are mainly constructed by synthetic and natural biopolymers. Natural polymers are preferred for edible hydrogel films and coating generation as they are non-toxic, biodegradable, and suitable to integrate with food substrate materials, which can improve sensory acceptance of dairy products [9,10]. Among natural biopolymers, chitosan and sodium alginate are particularly attractive for edible hydrogel film and coating applications due to their enhanced biocompatibility, non-toxicity, and film formation abilities [11].

Liposomes are spherical structures, composed of one or more phospholipid bilayers surrounding an aqueous core that has a membrane-mimicking structure [12]. Owing to their own structure, liposomes are capable of encapsulate both hydrophilic and hydrophobic compounds, protecting sensitive bioactive agents like enzymes, peptides and proteins, and drugs (for example, antibiotics) from environmental conditions, thus providing them with better stability [13].

There are studies related to hydrogel formation by using different biopolymers and encapsulation of lactase enzymes within these structures: the effects of pectin on lactose hydrolysis at various temperatures and pH levels [14], the impact of Arabic gum on the production of lactose-free or low lactose products [15], chitosan for producing milk with low lactose content [16], and the thermal stability and post-heat treatment activity of enzyme–biopolymer complexes formed with alginate [17] have been examined. Although these applications yield improved results against temperature and pH fluctuations, the large size, hydrophilicity, and weak mechanical resistance of hydrogels can lead to enzyme leakage from the matrix [18]. Encapsulation of enzymes within liposome particle vehicles represents a promising strategy to improve enzyme stability and functionality in food matrices [19]. Liposomal encapsulation can protect enzymes against adverse environmental conditions, such as pH fluctuations and enzymatic degradation, while allowing gradual enzyme release [20]. Edible hydrogel film and coating system applications on perishable foods are advocated to simultaneously improve shelf-life stability, modulate physicochemical properties, and provide targeted enzymatic activity aimed at lactose reduction [21].

In this context, this study aimed to develop and characterize lactase enzyme-encapsulated liposomes incorporated into chitosan-based (PCLLa) and polyelectrolyte chitosan–sodium alginate-based (CLLA) edible hydrogel film structures and coat them onto Perline Mozzarella cheese substrate. To the best of our knowledge, this is the first study on the coating of PCLLa and CLLA hydrogel-based films on Perline Mozzarella. The cheese is high-moisture Mozzarella and is produced using bovine milk. It has a small shape having around a 10 mm diameter and is consumed extensively as a table cheese [22]. Serving lactase enzyme together with the dairy product in the form of an edible coating is a novel approach that focuses on mimicking the normal digestion process to gain the benefits of consuming lactose that have been linked to influencing gut microbiota, improving mineral absorption and enhancing immune health. In addition, incorporating lactase enzyme-encapsulated liposome particles into edible hydrogel film coating structures further enhances this approach by manipulating lactase enzyme activity at the food surface, where lactose hydrolysis can occur in a controlled manner during storage and consumption. Also, edible hydrogel films and coatings offer a promising approach to modulate mass transfer processes and create a controlled microenvironment without compromising product integrity when they are applied onto the cheese surfaces.

In this form, dairy products are likely to be tolerated well in enzyme-efficient populations. In the current study, PCLLa and CLLA film structures were developed and characterised, and their effects on the physicochemical, sensory, and functional properties of the Perline Mozzarella cheese substrate during 60 days of shelf life were evaluated. Furthermore, in vitro release profile characteristics of the lactase enzyme-encapsulated liposomes-enriched edible hydrogel films coated on the cheese substrates was also assessed in the simulated digestion fluids mediums.

## 2. Results and Discussion

Lactase enzyme-encapsulated liposomes incorporated into chitosan-based (PCLLa) and polyelectrolyte chitosan–sodium alginate-based (CLLA) hydrogel film structures were applied to the Perline Mozzarella cheese substrate in a layer-by-layer manner.

### 2.1. Formation Mechanism of Lactase Encapsulation of Liposome Microspheres

**Figure 1:**
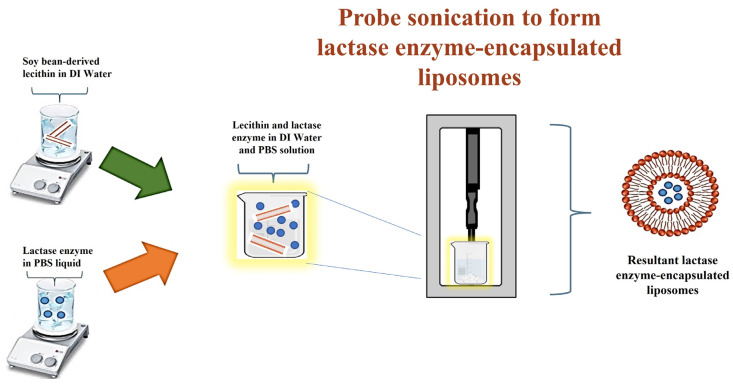
Preparation steps of lactase enzyme-encapsulated liposome microspheres. The addition of solutions into the beaker is indicated by two arrows: green for the lecithin solution and orange for the lactase enzyme solution. The blue spheres denote the lactase enzyme, while the red spheres denote lecithin.

**Figure 2:**
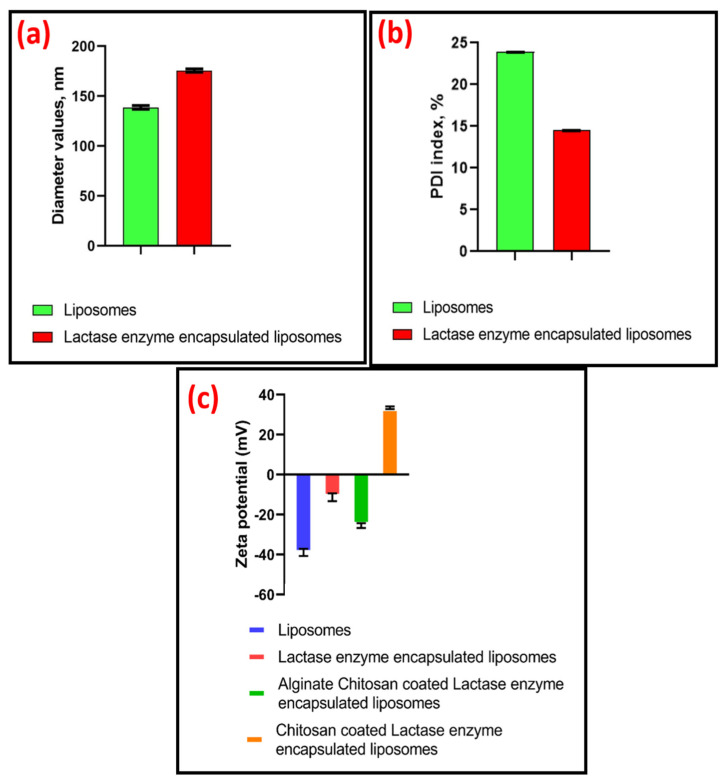
(**a**) Diameter (**b**) PDI, and (**c**) zeta potentials values of the liposome microspheres achieved.

Figure 1 illustrates the methodology by which the lactase enzyme-encapsulated liposome particles were prepared by using a probe sonication. In this route, phospholipids containing one or two fatty acyl chains spontaneously self-assemble into planar bilayer structures when dispersed in an aqueous medium. Upon the application of high-frequency acoustic energy via probe sonication, the amphiphilic molecules reorganise such that the hydrophobic acyl chains are oriented inward, while the hydrophilic head groups remain exposed to the surrounding water. The resulting disruption of the planar bilayers promotes their reassembly into closed liposomes, in which lactase enzyme is encapsulated in the aqueous core. The lactase enzyme has a hydrophilic protein structure with long chains of amino acids. The core compartment of the liposome is an aqueous environment in which the enzyme forms dipole–dipole interactions and hydrogen bonds through its polar amino acid side chains with water molecules that keeps the enzyme folded and active [23]. These liposome structures generally exhibit a finite period of physical stability, commonly described as kinetic stability; however, prolonged storage may lead to vesicle aggregation and a concomitant increase in particle size. Figure 2a presents that resultant liposomes were obtained successfully with an average particle size of 138.4 nm for bare liposomes and 175.6 nm for lactase-encapsulated liposomes with an encapsulation efficiency of >90%.

### 2.2. Physicochemical Properties of Two Different Liposome Microspheres

The influences of lactase enzyme encapsulation by probe sonication and layer-by-layer electrostatic interaction of polysaccharides on the particle size, polydispersity index (PDI) and zeta potential (ZP) of liposomes were studied. Average particle size and PDI were measured by dynamic light scattering (DLS). As seen in Figure 2a, enzyme encapsulation led to an enlargement in liposome diameter. PDI represents the size distribution of particles in a colloidal system. The recorded PDI data for liposomes were 20% whereas they were 10% for lactase enzyme-encapsulated liposomes (Figure 2b). This reduction in PDI may be because of the steric stabilisation that the system gains. The steric stabilisation prevents the liposomes from getting close to each other thus forming a more homogeneous structure [21,24]. For liposomes and liposome formulations, a PDI of 0.3 and below is considered to be acceptable and indicates a homogenous population of phospholipid vesicles [25].

Figure 2c represents the zeta potential values allocated for bare liposomes that were negatively charged as −39.2 mV, the lactase enzyme-encapsulated liposomes were negatively charged as −11.1, the PCLLa edible hydrogel film was positively charged as 33.3 mV, and the CLLA edible hydrogel film was negatively charged as −25.2 mV. Similar results were observed by Liu et al. (−25.2 ± 4.8 mV), after surface decoration of vitamin C-encapsulated liposomes with chitosan (1st layer) and alginate (2nd layer) [26]. Due to the repulsive forces, the zeta potential values greater than +30 mV and less than −30 mV are regarded as highly stable [13]. These results indicate effective film formation driven by electrostatic interactions between the liposomes, sodium alginate and chitosan polymers.

There are different techniques for liposome formation and encapsulation presented in the literature: conventional methods and novel approaches. Conventional methods are thin film hydration (Bangham method [27]), reverse phase evaporation (discovered by Szoka [28]), ethanol injection (developed by Batzri and Korn [29]) and dehydration–rehydration (developed by Kirby and Gregoriadis [30]) methods as presented in Table 1. The volatile organic solvents used in these processes are one of the drawbacks as their residues may remain in the structure and cause toxicity such as chloroform and ethanol. Moreover, in these methods, an additional step like ultrasonication is required to produce small, homogenised unilamellar liposomes as seen in Table 1. The microfluidic method, supercritical fluid and Mozafari [19] methods are represented as novel approaches compared to conventional methods [31]. Microfluidic technology offers a one-step encapsulation process that requires optimisation of microfluidic devices and the parameters like channel geometry, cross-sectional area, and the flow rate of liquid and gas phases to obtain high encapsulation efficiency [32]. But it is not easy to scale up; furthermore, it is time consuming to obtain a high amount of product compared to the probe sonication applied in this study. The liposomes, produced by the microfluidic method in Table 1, had particle sizes between 100 and 200 nm and the encapsulation efficiency was calculated between ~80 and 95% whereas the reported zeta potentials exhibit less stability. The supercritical CO_2_ (SP-CO_2_) method is a green synthesis approach that does not involve organic solvent or toxic chemicals. But in terms of this study, high pressure may damage the enzyme structure. The olive leaf extract mentioned in Table 1 was encapsulated by the SP-CO_2_ method with particle sizes ranging between 300 and 710 nm and 86% encapsulation efficiency. Another green synthesis technique is the Mozafari method [19], a single-step, solvent-free approach without any toxic risk that includes a pre-heating (40–60 °C) application which is a crucial factor for our study as the lactase enzyme was denaturated at 45 °C and above temperatures [33]. Moreover, the technique involves keeping liposomes in an inert atmosphere to improve stability. This method is suitable for large scale production and requires a special vessel designed by Mozafari [19]. Rosemary extract was encapsulated by the Mozafari method with 54.59% EE and this formulation exhibited a highest stability of −65.1 mV (Table 1). A higher zeta potential leads to higher electrostatic repulsion that reduces aggregation of liposomes due to the improvement in stability. In the presented study, probe sonication was applied for lactase encapsulation, and no toxic chemicals were incorporated. During probe sonication, as the probe temperature increases, an ice bath was used to prevent the denaturation of lactase enzymes and the pulse on–off time was also adjusted. According to the optimisation studies, high encapsulation efficiency was achieved (>90%) with a particle size of 175.6 nm.

**Figure 3:**
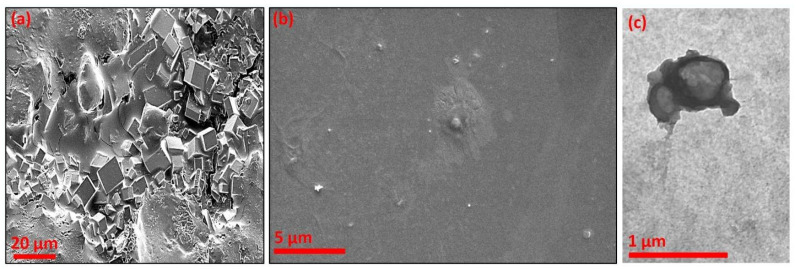
SEM capture of (**a**) the bare liposomes and (**b**) lactase enzyme-encapsulated liposome microspheres, and (**c**) TEM capture of edible hydrogel film-coated lactase enzyme-encapsulated liposome microspheres.

The SEM captures of bare liposomes and lactase enzyme-encapsulated liposomes, and TEM captures of edible hydrogel film-coated lactase enzyme-encapsulated liposomes are presented in Figure 3. As seen in Figure 3a, the bare liposomes appear like a cubic structure. In Figure 3b,c, the SEM and TEM pictures present that the enzyme-encapsulated liposomes tend to have a spherical shape. This transformation could indicate that the form of the cubic structure of bare liposomes is due to its larger overall surface area relative to a sphere form, with the cubic structure allowing for attachment of a greater number of targeting groups and the encapsulation of cargo ingredients [45].

**Figure 4:**
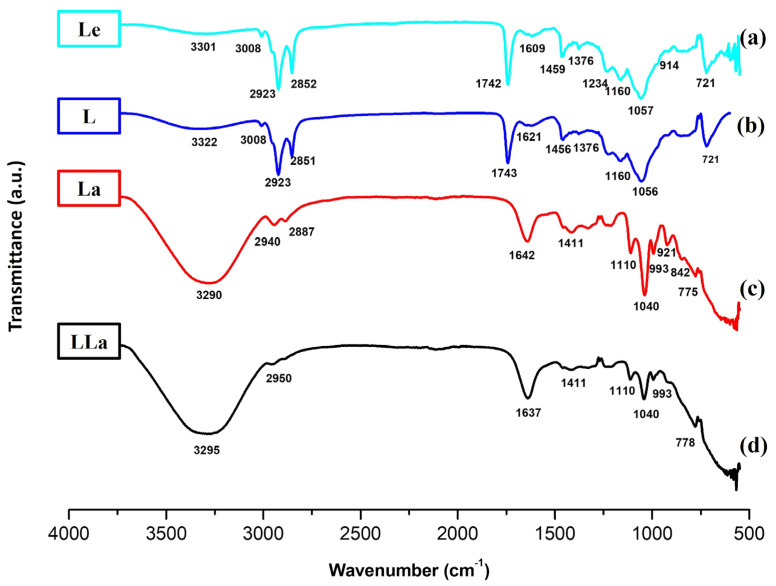
FTIR spectra for (**a**) lecithin, (**b**) the bare liposomes, (**c**) lactase enzyme, (**d**) lactase-encapsulated liposome.

The structures of lecithin, bare liposomes, lactase enzyme-encapsulated liposomes and lactase enzymes were analysed by FTIR spectrophotometry and can be seen in Figure 4. The typical phospholipid features of lecithin were a PO_2_ vibration between 1200 and 1145 cm^−1^, P–O–C vibrations between 1145 and 970 cm^−1^ and a C=O vibration between 1765 and 1720 cm^−1^ as seen in Figure 4a,b. Similar FTIR spectra are achieved by Machmudah et al. (2024) [46,47]. Particularly, the peak observed at 1742–1743 cm^−1^ is attributed to the C=O stretching vibration of the ester groups in the phospholipid molecules. In Figure 4c, a dominant band is seen at 1642 cm^−1^ which is the amide I band of the pure lactase enzyme. This signal of protein is more intense than phospholipids’ ester signal so that it can dominate this region in Figure 4d. Moreover, the shift from 1642 cm^−1^ in the pure lactase enzyme to 1637 cm^−1^ in LLa demonstrates that intermolecular hydrogen bonds formed between the amino groups of enzymes and the polar head groups of phospholipids. This interaction confirms that the enzyme is stabilised maintaining its structural integrity within the liposomal structure. After liposome preparation, the bare liposome spectrum exhibited the characteristic bilayer signals of asymmetric/symmetric –CH_2_ stretching bands at around 2922 and 2853 cm^−1^ and the ester carbonyl (C=O) stretching band at around 1742 cm^−1^. Free lactase enzyme (La) presented protein-specific absorptions, notably a broad N–H/O–H band (~3285 cm^−1^) and a distinct amide I band at ~1642 cm^−1^, with additional bands in the 1462–1411 cm^−1^ region and strong signals in the 1109–1039 cm^−1^ region attributed to C–N/C–O vibrations. The band at 3500–3200 cm^−1^ was associated with hydrogen bond stretching vibrations of N–H and O–H which got broader and wider by the encapsulation of the lactase enzyme because of enhanced hydrogen bonding interactions in Figure 4c. Similar FTIR spectra are achieved by Zang et al. [47]. Importantly, the lactase-encapsulated liposome (LLa) showed the coexistence of lipid and protein features; the lipid fingerprint band near ~1051 cm^−1^ was retained while a clear protein contribution appeared with the amide I band shifting from ~1642 cm^−1^ (free enzyme) to ~1633 cm^−1^ after encapsulation as seen in Figure 4d. This downshift, together with changes in the headgroup/fingerprint region (~1160–1039 cm^−1^), suggests non-covalent interactions between lactase functional groups and phospholipid moieties within the liposomal microenvironment.

### 2.3. Coating Formation Mechanism of Polyelectrolyte Polymers-Based Edible Hydrogel Film Structures on Cheese Product

**Figure 5:**
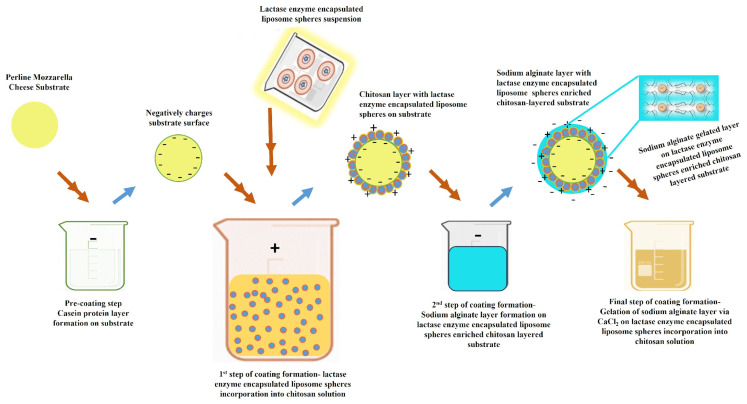
Schematic representation of the coating process steps of lactase enzyme-encapsulated liposome spheres-enriched edible hydrogel film on cheese substrate. The red arrows indicate the addition of cheese or solutions into the beaker, whereas the blue arrows represent the resulting forms.

The schematic coating formation mechanism of the polyelectrolyte polymers-based edible hydrogel film coating achieved is illustrated in Figure 5. The coating methodology for layer-by-layer self-assembly of alginate and chitosan polyelectrolyte biopolymers was utilised on alternate deposition of opposite charges, generated through the weak electrostatic interactions between alginate and chitosan layers. The amphoteric structure of the casein protein in cheese had the ability to become positively or negatively charged by adjusting its isoelectric points. As a part of the coating process, initially the substrate cheese material surface was negatively charged as −23 mV zeta potential value, by immersing it in PBS adjusted to pH 7. Lu et al. explained that the negative surface of cheese can successfully form electrostatic bonding interactions with chitosan polymers [48]. Before the coating process, lactase-encapsulated liposome solution was incorporated into the chitosan solution to achieve interactions between liposomes and chitosan molecules. The liposome’s surface is composed of polar head groups of lecithin that carry negatively charged phosphate groups. Chitosan is a polycationic polymer and in acidic environments its amino groups are protonated which leads to a strong positive charge. As a result, an electrostatic interaction occurs between the liposome surface and chitosan chains that form a protective layer [49]. Furthermore, both the amino groups and hydroxyl groups of chitosan molecules form hydrogen bonds between polar head groups of phospholipids which leads to a stabilised coating. The electrostatic deposition on the cheese substrate surface and between the polyelectrolyte lactase enzyme-enriched chitosan and sodium alginate layers was achieved by positively charged chitosan and negatively charged sodium alginate during the dip coating process [50]. Electrostatic deposition on the cheese surface can be formed by allowing negatively charged casein on the cheese surface and positively charged ammonium groups of chitosan. Similar electrostatic deposition on the chitosan layer was built up due to the protonated amino groups (in the acidic environment) of the chitosan layer and negatively charged carboxylic groups of sodium alginate that undergo an ion-ion electrostatic interaction and form a strong, water-insoluble polyelectrolyte complex. Moreover, the oxygen atoms of carboxylic groups of alginate and amino groups of chitosan form hydrogen bonds that effect the mechanical strength and water absorption capacity of the film structure. At the end of the coating process calcium chloride was added as a crosslinking agent to obtain gelation by crosslinked hydrogel networks. The ionic crosslinking forms between G blocks of sodium alginate (sodium alginate has L-guluronic acid (G) and D-mannuronic acid (M) residues) and divalent Ca^2+^ ions known as the Egg-Box model, which is a three-dimensional, water-insoluble, hydrogel structure [51]. Ca^2+^ has two positive charges that give it the opportunity to bind with two adjacent alginate chains at the same time and form bridges. In addition, ion exchange occurs via the displacement of divalent calcium ions (Ca^2+^) with monovalent sodium ions (Na^+^). This exchange transforms water-soluble sodium alginate to water-insoluble calcium alginate. In acidic environments, the gel structure remains stable but in basic environments it tends to dissolve; that is the main goal of this study.

### 2.4. Characterisation Results of Polyelectrolyte Polymers-Based Edible Hydrogel Film Structures on Cheese Product

**Figure 6:**
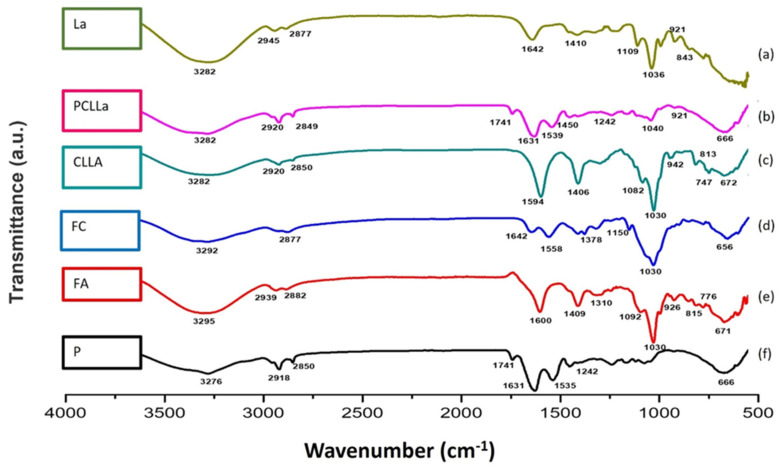
FTIR spectra of (**a**) lactase enzyme (La), (**b**) lactase enzyme-encapsulated liposomes-enriched chitosan layer formed cheese (PCLLa), (**c**) sodium alginate and chitosan polyelectrolyte polymer edible hydrogel film (CLLa), (**d**) only chitosan edible hydrogel film layer (FC), (**e**) only sodium alginate edible hydrogel film layer (FA) and (**f**) Perline Mozzarella cheese substrate (P).

Fourier-Transform Infrared (FTIR) spectroscopy was used to identify the interactions between molecules and the chemical composition of the structure, based on the absorption of infrared light. According to Figure 6b, sample PCLLa’s peak that occurred at 1410 cm^−1^ is attributed to the carboxylate (–COO) ions presented in lactase enzyme structure. The FTIR spectrum of saccharides-incorporated liposomes contained three groups of characteristic peaks at 1384 cm^−1^ (deformation of C–H bonds), 2854 cm^−1^ and 2925 cm^−1^ (CH_2_ stretching vibration) [52]. In addition, the chemical bonding structure peak of cheese was observed at 1631 cm^−1^ and 1535 cm^−1^.

In the FTIR spectra of CLLA films presented in Figure 6c, the –NH stretching of the amine group at 1642 cm^−1^ in chitosan disappeared; that may be the result of the interaction between –NH3+ (amine group of chitosan) and –COO ions (carboxylic group of sodium alginate). The broad band at around 3290 cm^−1^ became weaker because of the free O=H stretching that decreased due to the interactions between the crosslinker and –OH groups in chitosan or sodium alginate chains. (Both FTIR spectra comparing pure chitosan polymer and the chitosan films fabricated are provided in Figure S1 and the results of FTIR in Figure S2 are also presented with a comparison of the pure sodium alginate polymer and the sodium alginate film formed.) The peak observed at 1558 cm^−1^ that corresponds to amide II stretching disappeared confirming that the –NH_2_ group of the chitosan chain participates in the crosslinking process. As seen in the FTIR results most of the characteristic peaks that belong to chitosan and sodium alginate are preserved and some have disappeared because of crosslinking in the layer-by-layer structure.

The absorption band that occurred at the fingerprint region between 920 and 1100 cm^−1^ corresponds to C–O–C skeletal stretching. The sharp peak that occurred at 1030 cm^−1^ represents that the carboxylic groups found in the main skeletal structure were preserved. C–H stretching in the mannuronic unit of sodium alginate was observed at 925 cm^−1^. It is clearly seen that the characteristic peaks of sodium alginate got more intense in edible hydrogel film formation because of stretching and vibrations. The peak observed around 2927 cm^−1^ corresponds to C–H stretching [53] and the vibrations between 1600 and 1400 cm^−1^ were asymmetric and symmetric stretching of carboxylate ions (–COO–) of sodium alginate [54].

#### 2.4.1. Structural and Morphological Characterisation Results of Polyelectrolyte Polymers-Based Edible Hydrogel Film Structures on Cheese Product

**Figure 7:**
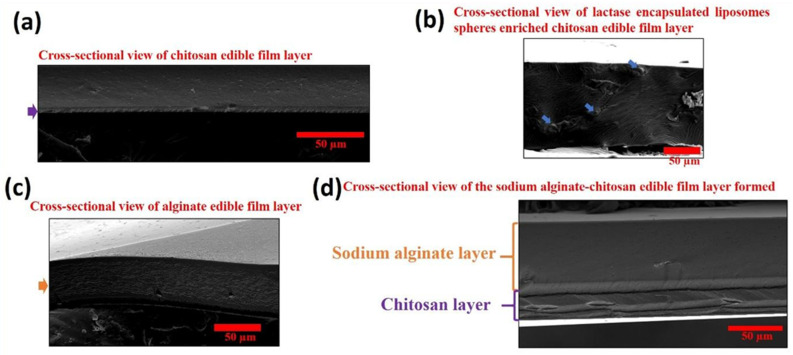
Cross-section SEM captures of (**a**) only chitosan edible hydrogel film (purple arrow presenting thickness of the coating layer), (**b**) the lactase enzyme-encapsulated liposomes-enriched chitosan edible hydrogel film (PCLLa) (blue arrow presenting lactase enzyme encapsulated liposome microspheres), (**c**) only sodium alginate edible hydrogel film (orange arrow presenting thickness of the coating layer), and (**d**) the sodium alginate–chitosan polyelectrolyte polymer edible hydrogel film (CLLA) formed by using a dip coating method.

The SEM microscope images describing the surface morphology of edible hydrogel films are shown in Figure 7. The chitosan edible hydrogel film (Figure 7a) had a homogenous surface without pores whereas in Figure 7b lactase-encapsulated liposomes appeared as a dotlike shape, incorporated in the (PCLLa) structure. The surface scanning results in Figure 7c indicated that CaCl_2_ formed a porous structure in sodium alginate edible hydrogel films through the crosslinking process. The sharp transition between two layers (upper layer sodium alginate and bottom layer chitosan) in Figure 7d confirms that the edible coating film formed by a dip coating method with a layer-by-layer electrostatic interaction was applied successfully. The thickness of edible hydrogel films was recorded as ~5 µm for chitosan films, ~70 µm for sodium alginate film and ~90 µm for CLLA edible hydrogel film structures as can be seen in Figure 7. Incorporation of a crosslinking agent could create a porous structure that leads to increments in thickness. Alternatively, the materials could be made porous using bubble forming technologies using suitable processes [55].

The mechanical properties of biopolymer films were associated with intermolecular and intramolecular interactions within the polymer matrix. The average tensile strength values were obtained as approximately 9.102 MPa, 2.029 MPa and 27.35 MPa for the PCLLa edible hydrogel film, only sodium alginate edible hydrogel film and CLLA edible hydrogel film, respectively. Sodium alginate edible hydrogel films represented more flexible structures than chitosan-based edible hydrogel films. Based on the literature, the improved tensile strength of edible hydrogel films formed by using a dip coating method could be associated with the hydrogen bonds formed between the hydroxyl groups of sodium alginate and ammonium groups of chitosan [56]. The elongation at break values represented the ability of a film to stretch; the results evaluated were approximately 7.6%, 13.23% and 8.62% for the PCLLa edible hydrogel film, sodium alginate edible hydrogel film and CLLA edible hydrogel film, respectively [53]. The Young’s Modulus values of the PCLLa edible hydrogel film, the sodium alginate edible hydrogel film and the CLLA edible hydrogel film were recorded as approximately 157.25 MPa, 21.57 MPa and 514.16 MPa, respectively. The results indicate that sodium alginate edible hydrogel film showed more flexible behaviour than the other films. This, in effect, supports the tensile strength results obtained. The greater Young’s Modulus means the higher stiffness and resistance to elastic deformation under load [57].

#### 2.4.2. Results of Shelf-Life Analysis

##### Results of Chemical Properties Analysis (pH Measurements and Dry Matter Determination Measurements)

The changes in the pH values of the Perline Mozzarella cheese substrate (control) and coated cheese samples (PCLLa and CLLA) during storage are presented in Table 2. During storage, significant alterations in pH were observed among the samples and storage times (*p* < 0.05). The control sample showed a slight increase in pH and remained relatively stable throughout the storage period. This increment may be associated with proteolytic activity and the accumulation of alkaline by-products in the uncoated cheese matrix. The PCLLa exhibited a fluctuating but overall decreasing pH trend during storage. Although a temporary increase was observed on the 15th day, a significant reduction in pH occurred from the 30th day onward (*p* < 0.05), reaching the lowest value on the 60th day. This behaviour can be attributed to the acidic nature of chitosan solutions and their ability to restrict the diffusion of alkaline metabolites from the cheese surface. The lowest pH values were observed in chitosan–sodium alginate bilayer-coated samples (CLLA) throughout the storage period, suggesting that the calcium-crosslinked sodium alginate outer layer may have restricted oxygen diffusion and limited gas and metabolite exchange while enhancing the retention of acidic components, thereby promoting a more acidic surface environment. A similar reduction was recorded by Liu et al. in the pH (from 5.77 to 5.49) of the chitosan-pectin complex incorporated into the cheese [58] matrix.

The effects of edible coatings on the total solids content of cheese samples during refrigerated storage are presented in Table 3. On day 0, no significant differences were observed among the samples (*p* > 0.05), with all treatments exhibiting identical total solids values (43.0%), indicating a homogeneous initial composition prior to coating application. During storage, the control sample showed relatively stable total solids values with a slight but significant increase observed on the 15th, 30th, and 45th days compared to day 0 (*p* < 0.05). However, a significant decrease was recorded on the 60th day, suggesting structural changes such as proteolysis that expresses the breakdown of proteins into smaller peptides leading to an increase in water-binding capacity in the uncoated cheese matrix. The recorded decrease may also hypothesise the weakening of the protein matrix and increased water retention as well due to microbial growth. Both the chitosan-coated and chitosan-sodium alginate-coated samples showed a decrease up to the 60th day which may be related to water binding at the surface of the liposomes [59]. The PCLLa showed significantly lower total solids compared to the control group on the 60th day. The observed reduction may be attributed to the hygroscopic nature of chitosan-based coatings, which can facilitate moisture absorption over time. Al-Moghazy et al. reported that chitosan coating did not affect the dry matter content of Karish cheese [60]. The mentioned difference between the two studies may be attributed to the influence of vacuum packaging on water loss [60]. A significant decrease was observed in the CLLA samples continuing through days 30 and 45 (*p* < 0.05) which may be as a result of the high water absorbing capacity of sodium alginate. Notably, on the 60th day, the total solids content of CLLA samples increased significantly and reached values comparable to the control group (*p* > 0.05). This behaviour may be related to the deviations in the pH from the isoelectric point of casein. At pH values above this point, casein exhibits increased water-holding capacity that can contribute to changes in the total solid content. But the total solid content results on the 60th day are still in the acceptable range of 40–46%.

##### Results of Colour Analysis

Colour is an important quality indicator for cheese that directly influences consumer acceptability as it is generally linked to deterioration. The effects of edible coatings during storage on cheese parameters of L* (lightness), a* (red-green coordinate) and b* (yellow-blue coordinate) values are shown in Table 4. The a* values of all samples were negative during the storage period, indicating a predominance of green tones typical for fresh dairy products. Similarly, negative a* values were reported in alginate-coated Ricotta cheese [61]. The control and PCLLa samples exhibited a gradual decrease in a* values, becoming significantly more negative by the 60th day (*p* < 0.05). This shift toward greener tones may be associated with moisture loss and surface dehydration. The CLLA sample exhibited significantly higher lightness values (L*) compared to both the control and PCLLa samples (*p* < 0.05) likely due to improved moisture retention. The performances of chitosan and sodium alginate (separately) edible coatings on Mozzarella cheeses were investigated by Zhong et al. [62]. The lightness values decreased for both chitosan and alginate-coated cheeses. A similar decrease was recorded in this study in PCLLa but in CLLA there was no significant difference in L* values suggesting that the multilayer chitosan–sodium alginate coating effectively preserved surface brightness which may be due to the physicochemical changes occurring on the cheese surface, such as moisture redistribution or alterations in pigment stability. The CLLA sample maintained relatively stable a* values throughout storage, with no statistically significant differences observed between sampling days (*p* > 0.05). Notably, at day 60, the CLLA sample displayed significantly higher (less negative) a* values compared to the control and PCLLa (*p* < 0.05), indicating better colour stability. The a* values of Mozzarella cheeses that were coated by chitosan and alginate separately exhibited a decrease for both, as depicted by Zhong et al. [62], whereas our results demonstrated that the chitosan-sodium alginate multilayer coating maintained a more stable colour profile. The increase in b* values (yellowness) is commonly associated with lipid oxidation and rancidity [63]. The b* values, representing yellowness, increased significantly in all samples during storage (*p* < 0.05). This may be attributed to the light scattering effects and increased surface smoothness provided by the multilayer coating structure as no rancid taste was perceived in the cheese samples. Overall, the CLLA sample demonstrated more uniform and controlled changes over time, indicating that the combined chitosan-sodium alginate coating improved colour homogeneity and visual quality.

##### Results of Microbiological Analysis

Table 5 represents the total viable counts of bacteria and yeasts. The total bacterial counts of all samples were identical (7.94 log CFU/g) at the beginning which leads to comparable microbiological analysis for the Perline Mozzarella cheese substrate (control) and coated (PCLLa and CLLA) ready to eat cheeses. Significant differences (*p* < 0.05) were observed among the samples stored at +4 °C for 60 days. According to the total bacterial counts, there was a stable trend in the control sample after a slight decline in the first 15 days. Despite this stable trend, the control sample statistically exhibited lower stability during storage time among the other samples. Although the number of viable colonies of the PCLLa sample was decreased in 15 days, a gradual increase was observed until the 60th day. Lu et al. reported that lactic acid bacteria (LAB) ferment the residual lactose in cheese and form lactic acid that leads to an increase in acidity. When the acidity reaches a certain level, acid production stops. Because of the already formed acidic environment, the bacterial growth might be inhibited. But after LAB stops working, mould and yeast start to act and produce alkaline metabolites [48]. Chitosan exhibits an antimicrobial effect by interacting between its amino groups (positively charged) and microbial cell membrane (negatively charged) [64]. According to the results, the antimicrobial activity of chitosan alone was not effective enough to suppress bacterial growth under the applied conditions. This result may be explained by the limited barrier properties of chitosan, a reduction in antimicrobial effect during storage time or a possible contamination related with the process. The CLLA sample exhibited a greater decrease in total bacterial counts in the early storage period in which significantly lower values (*p* < 0.05) were recorded compared to the PCLLa and control samples. The synergistic activity of sodium alginate and chitosan enhanced the antimicrobial efficiency and barrier properties as well. Toward the 60th day the viable count results were increased with a decrease in antimicrobial effect as seen in PCLLa. In this group no significant effect against yeast growth was obtained, demonstrating that this combination was more effective against bacteria than yeast growth. The statistically lower yeast counts in the PCLLa sample indicate the more inhibitory effects of chitosan against yeasts during storage.

##### Results of Sensory Analysis

Sensory scores of the resultant cheese samples are presented in Table 6. The highest scores achieved with the CLLA samples correspond to better visual appearance and colour throughout the whole storage time (after 60 days) (Table 6). Bitterness rating values were constantly high for all samples indicating that there was no bitter taste (Table 6). In Table 6, odour, saltiness and sourness values of the CLLA had high scores with no significant difference among all samples. Overall acceptability scores remained within an acceptable range across the entire storage period for all samples. In the literature, the appearance and colour parameters of food products can be used to describe the visual acceptance of the dairy product by consumers [5,65]. The sensory analysis scores of “Göbek Kashar” cheeses of uncoated, only chitosan-coated and fish oil-fortified chitosan-coated samples were evaluated by Yangilar et al. [66]. The only chitosan-coated sample was the most preferred among all with the highest overall acceptability. Layer-by-layer self-assembled chitosan and flaxseed gum-coated Mongolian cheese’s sensory analysis also demonstrated that chitosan does not influence the sensory attributes negatively [48].

##### Results of in Vitro Lactase Enzyme Release Behaviour Analysis

**Figure 8:**
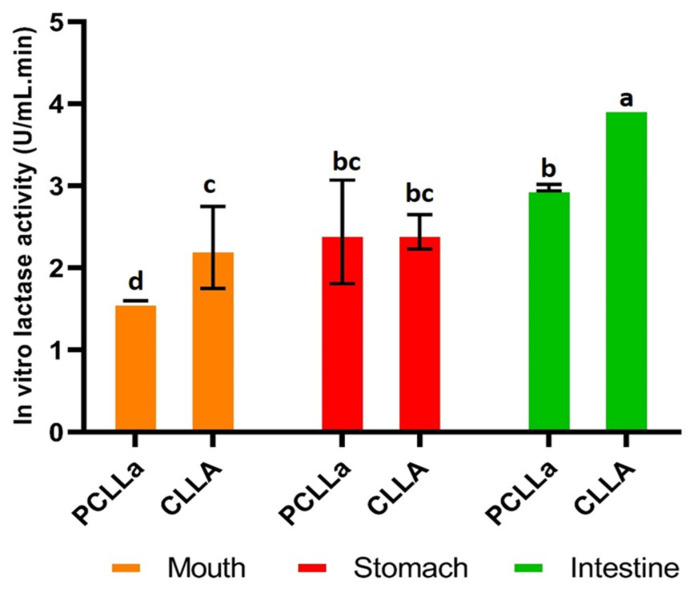
In vitro release results of the cheese samples during shelf-life (Values are presented as mean ± standard deviation. Different lowercase letters indicate statistically significant differences among all groups according to Duncan (*p* < 0.05)).

Lactase enzyme release profile values from the liposomes produced that were exposed to simulated saliva fluid (SSF), simulated gastric fluid (SGF) and simulated intestinal fluid (SIF) are shown in Figure 8. In the SSF environment (pH value: ~6.7), the average lactase enzyme release amount from the PCLLa was 1.60 U mL^−1^ min^−1^ and the CLLA was 2.25 U mL^−1^ min^−1^. These results indicated that a lactase enzyme presented slow-release behaviour for the PCLLa samples compared to the CLLA samples because the chitosan may become less protonated and poorly soluble, which is tending its form to remain as a denser film. Similarly, the carboxyl groups of sodium alginate were largely deprotonated, which can promote hydration and swelling of the outer layer [67]. However, the main driver in the mouth could be mechanical disruption and dilution (chewing and saliva flow) and it may be more influential than chemistry because the contact time took only 5 min.

In the SGF environment (pH value: 1–3), the average lactase enzyme release amount from the PCLLa and CLLA was 2.44 U mL^−1^ min^−1^ after two hours of incubation time (See Figure 8). These results presented that the release behaviour of the CLLA samples depicted was showing slow release. There could be two causes to unveil the slow-release behaviour of these samples. Firstly, in acidic media, pH < pKa of sodium alginate edible hydrogel film, which leads to protonation of carboxylic groups, solubility decreases and Ca–alginate networks typically adopt a denser structure with reduced permeability and limiting acid penetration. Secondly, the liposomes could resist the acidic environment of the stomach [68]. On the other hand, the average lactase enzyme release behaviour from the PCLLa presented a remarkable increase in the SGF environment compared to the SSF medium. In these samples, amino groups of chitosan could become extensively protonated as pH < pKa, which generally promotes hydration and swelling and may partially increase coating solubilization, which may demonstrate with a significant increase in the release profile between simulated saliva fluid and gastric fluid.

In the SIF environment (pH value: ~6.7), the average lactase enzyme release amount from the PCLLa was 2.98 U mL^−1^ min^−1^ and the CLLA was 3.96 U mL^−1^ min^−1^ for two hours of exposure under jejunum conditions (Figure 8). These results demonstrated that the release behaviour of the PCLLa samples depicted was displaying slow regime because of the deprotonation of the chitosan polymer and thus becoming a less soluble form which could reduce further dissolution. On the other hand, the average lactase enzyme release behaviour from the CLLA samples presented a remarkable increase in the SIF environment compared to the other mediums. This could be as a result of the sodium alginate carboxyl groups that became deprotonated, which leads to increased swelling and higher permeability of the calcium–sodium alginate network, which may progressively loosen the CLLA sample’s structure and increase its permeability. Moreover, an increase in the lactase enzyme release profile in the CLLA samples also caused a rapid change in the pH of the SIF environment which may affect the ionisation of phospholipid head groups in liposome structures and increase membrane fluidity that could disrupt them and thus an increase in the release of lactase enzymes [34].

Overall, the cumulative release of lactase enzymes from the PCLLa samples was 7.02 U mL^−1^ min^−1^ and from the CLLA samples was 8.65 U mL^−1^ min^−1^ (Figure 8). These results indicated that the lactase enzyme release throughout the digestion system displayed the highest amount in the SIF medium as expected. Tan et al. designed an oral delivery system based on hydroxy-α-sanshool-encapsulated liposomes coated with chitosan and alginate using a layer-by-layer coating method [69]. Faster release was observed in uncoated liposomes whereas alginate–chitosan coating exhibited a controlled drug release [69]. Similar controlled release behaviour of alginate–chitosan was recorded by Hu et al. for the liposomal delivery of apple peel polyphenols [34] and by Liu et al. who studied the environmental stress stability of vitamin C-encapsulated liposomes coated with alginate-chitosan [26] in the digestive system. The variance analysis demonstrated that the fluctuated pH in the digestion system may remarkably influence the enzyme release behaviour which confirms a release profile change in the lactase enzyme for both the PCLLa and CLLA samples.

Uncontrolled enzyme release is a significant point in food technology and especially in pharmacology as it may cause the loss of functionality of enzymes or the loss of yield. Specifically, regarding the lactase enzyme, when it releases into the acidic environment of the stomach (pH 1–3), the amino acids at the active site that are responsible for catalysis lose their ionic state and, as a result, lactase cannot bind to its substrate (lactose). Moreover, the proteolytic enzyme, pepsin, denatures the lactase structure, so the three-dimensional conformation damages and the enzyme lose its functionality. These effects are predominantly observed when lactase is administered as uncoated preparation. In this study, although lactase release was determined both in the mouth and stomach, the highest amount of release was observed in the CLLA formulation in the small intestine.

## 3. Conclusions

Apart from the commercially available products for lactose-intolerant individuals, a novel delivery system for lactase enzymes, in the form of an edible coating on Perline Mozzarella cheese, was studied to gain the benefits of lactose digestion in the small intestine. The research focused on enzyme encapsulation, the in vitro release behaviour of edible coatings and the effects on shelf life. Lactase-encapsulated liposomes-enriched chitosan edible hydrogel film (PCLLa) and lactase-encapsulated liposomes-enriched polyelectrolyte chitosan and alginate edible hydrogel film (CLLA) coatings were successfully formed on Perline Mozzarella. The encapsulation process was achieved via probe sonication and edible hydrogel film coating was achieved via layer-by-layer electrostatic interactions of biopolymers by using a dip coating method. The encapsulation of the lactase enzyme in liposomes and their structural morphology was confirmed by SEM and TEM analysis and the chemical–physical structural forms of liposomes were verified by using FTIR, zeta potential and DLS measurements. The liposomes had an average particle size of 138.4 nm for bare liposomes whereas lactase-encapsulated liposomes had a 175.6 nm particle size. The lactase enzyme encapsulation efficiency was determined as >90%. The shelf-life analysis of CLLA demonstrated better results up to the 60th day in terms of physical, chemical and sensory properties with the highest brightness in colour of 86.26, 42.60% in total solids content and 5.48 in pH. The digestion conditions significantly affected the release of the lactase enzyme, and the highest amount was obtained as 3.96 U mL^−1^ min^−1^ in CLLA in the SIF medium as expected. It is imperative that orally administered formulations containing lactase enzymes should protect the enzyme from the pH fluctuations that occur in both oral and gastric fluids, ensuring its intestinal delivery. A limitation of the study is the cumulative release of the lactase enzyme before it is delivered to the small intestine, but the results demonstrate that the CLLA formulation overcame this possibility in a better manner than PCLLa (2.98 U mL^−1^ min^−1^ in SIF). This study opens a perspective in the dairy industry. This is a prototype study addressing the needs of the lactose-intolerant population; nevertheless, this approach could be used to deliver other protein-based medicines to the specific part of the intestine. Future research will focus on broadening these applications on the other dairy matrices for lactose-intolerant people.

## 4. Materials and Methods

### 4.1. Materials

The encapsulation excipient enzyme, lactase (Beta-galactosidase, 6500 LAU/g), and digestive enzyme amylase (300 AGU/mL) were received as a gift from Novonesis company, Istanbul, Türkiye. Commercial phosphate-buffered saline (PBS) solution (Sigma Aldrich, Rockville, MD, USA) was used for making an enzyme ready for liposomal encapsulation. L-α-Phosphatidylcoline (lecithin from Soybean, Type IV-S, Sigma Aldrich, Rockville, MD, USA) was used for liposome production, analytical-grade sodium alginate (Sigma Aldrich, Rockville, MD, USA) and chitosan (≥75% degree of deacetylation, CAS number: 9012–76-4, Sigma Aldrich, Rockville, MD, USA) were employed for polyelectrolyte edible hydrogel film coating layer forming, and calcium chloride dehydrate (CaCI_2_) for physical crosslinking of sodium alginate layer was purchased from Sigma-Aldrich, Rockville, MD, USA. Food-grade glacial acetic acid was bought from Sigma-Aldrich, Rockville, MD, USA. This type of acetic acid is suitable to use as an edible film ingredient [70]. Distilled water used in this experimental work was filtered by a Millipore device (Arium 611UV, Sartorius AG, Göttingen, Germany) to prepare liposome, sodium alginate and CaCI_2_ solutions. Commercially available Perline Mozzarella shaped cheese substrates were obtained from a local shop in Hatay, Türkiye, and they were approximately 4 g each, semi-hard and had a salty taste. Tryptic soy agar (TSA) and OGYE selective supplement to prepare oxytetracycline glucose yeast agar (OGYA) medium were supplied from Merck, Darmstadt, Germany. Digestion model enzymes were purchased from Yenilab company (Kartepe, Kocaeli, Turkey) to perform in vitro analysis experiments.

### 4.2. Preparation of Lactase-Encapsulated Liposome Microspheres and Their Characterisation (EE, DLS, Zeta Potential, SEM, TEM, FTIR)

Lactase enzyme solution was prepared by stirring 20% (*v*/*v*) lactase and 60% *v*/*v* PBS at room temperature (20 °C ± 5). Liposome solution was formed with a handshake using 8% (*w*/*v*) lecithin in distilled water. Both solutions were mixed with continuous stirring at 30 °C for 15 min to encapsulate lactase enzyme into liposomes by using ultrasonic probe sonication method (Nanografi Nano Technology, Ankara, Türkiye) at 70% amplitude for 5 min with 5 cycles [31]. The encapsulation efficiency was determined by removing the free enzyme from liposome dispersions by centrifugating at 9000 rpm for 15 min at 4 °C in a Hettich-Universal 320R series ultracentrifuge (Andreas Hettich GmbH & Co., Ltd. KG, Tuttlingen, Germany). The amount of enzyme in supernatant was defined spectrophotometrically at 420 nm. Encapsulation efficiency (EE%) was calculated as follows:

(1) $$EE,\%=\frac{m_{i}-m_{sup}}{m_{i}}\;\times \;100$$

where m_i_ is the initial amount of lactase enzyme used for liposomal encapsulation and m_sup_ is the amount of enzyme determined in supernatant. The average particle size, size distribution (PDI value), stability and surface charge properties of the liposomes were evaluated using dynamic light scattering (DLS, Malvern Instruments Limited, Nano series, Malvern, UK). Detailed morphological imaging and dimensional features of the liposomes were unveiled by using scanning electron microscope (Philips XL 30, SFEG, York, UK) with the accelerating voltage of 5kV and transmission electron microscope (ESOGU, Hitachi Regulus 8230, Tokyo, Japan). FTIR analyses were performed to define the possible alterations both in functional group and fingerprint reagent of the lactase enzyme-encapsulated and bare liposomes achieved within a spectrum from 4000 to 500 cm^−1^ wavenumber (PerkinElmer Spectrum One Series FTIR Instrument version 5.0.1, Springfield, IL, USA).

### 4.3. Development of Polyelectrolyte Polymers-Based Edible Hydrogel Film Coatings on Cheese Product

Two types of edible hydrogel film coatings were developed: lactase-encapsulated liposomes-enriched chitosan (PCLLa), and lactase-encapsulated liposomes-enriched polyelectrolyte chitosan and sodium alginate (CLLA). For the development of PCLLa coatings, chitosan solution (1.5%, *w*/*v*) was prepared in 0.5 M acetic acid at 60 °C. Subsequently, the lactase-encapsulated liposomes (prepared as described in Section 4.2) were gradually incorporated in the chitosan solution with a ratio of 1:1 under continuous stirring at room temperature. Film coating was performed with a layer-by-layer electrostatic interaction by using a dip coating method. Initially, the cheese substrates were immersed into the PBS solution (pH 7.4) for 5 s to change their surface charge, negatively. Then, the cheese substrates were immersed in the prepared lactase-encapsulated liposomes-enriched chitosan solution for 2 min. Subsequently, the samples were taken out to drain and dry. The CLLA hydrogel film coating was prepared in the same manner with PCLLa film with an additional step which included immersion of the dried PCCLa samples in 2% (*w*/*v*) sodium alginate for 2 min and then in 2% (*w*/*v*) CaCI_2_ solution for 2 min to physically crosslink sodium alginate layer [71]. After coating process was performed, the samples were removed to drain and dry for 5 min. The coated cheese substrates were vacuum packed and stored at 4 °C until further characterisation and analysis of shelf life. All the solution preparation and coating processes were performed under sterile conditions.

### 4.4. Characterisation of Polyelectrolyte Polymers-Based Edible Hydrogel Film Coatings on Cheese Product

#### 4.4.1. Structural and Morphological Characterisation (SEM, TEM, FTIR and Mechanical Analysis)

The morphological capturing and structural features of PCLLa or CLLA coatings on Perline Mozzarella cheese were evaluated using a scanning electron microscope (Philips XL 30, SFEG, York, UK) with the accelerating voltage of 5kV and a transmission electron microscope (Hitachi Regulus 8230, Tokyo, Japan). The thickness of these edible hydrogel film coatings was measured by using digital calliper. Tensile strength, elongation at break point and Young’s Modulus values were obtained by examining mechanical tensile strength test (Instron 5569, Norwood, MA, USA). FTIR spectra of the all edible hydrogel film coatings and the Perline Mozzarella cheese substrates were achieved to identify the interactions between molecules and the chemical composition of a structure based on the absorption of infrared light within a spectrum from 4000 to 500 cm^−1^ wavenumber (PerkinElmer Spectrum One Series FTIR Instrument version 5.0.1, Springfield, IL, USA).

#### 4.4.2. Shelf-Life Analysis

Post-polyelectrolyte polymers-based edible hydrogel film coatings protection in stability during the 60 days of storage at 4 °C was assessed by examining pH and dry matter determination measurements, colour and sensory analysis, microbiological evaluation, and in vitro lactase enzyme release.

##### Chemical Properties Analysis (pH Measurements and Dry Matter Determination Measurements)

Changes in acidity of the samples, the control material, PCLLa and CLLA were recorded using a calibrated digital pH meter (Microprocessor pH 211, HANNA Instruments Inc., Winsocket, RI, USA) at room temperature. Dry matter analyses were performed by drying the samples at 102 °C according to the IDF:2004 [72].

##### Colour Analysis

To observe colour change in the Perline Mozzarella cheese samples having 60 days storage, colour parameters, including L (lightness), a* (redness), and b* (yellowness), were measured using a colour analyzer (Lovibond RT300 Series Reflectance Tintometer, Salisbury, UK). Each measurement was replicated four times to obtain an average value.

##### Microbiological Analysis

The microbial load was determined in the cheese samples (control, PCLLa, CLLA) following the storage period by monitoring total bacterial count and total yeast count. In the analysis, 10% *w/v* cheese samples were mixed with a sterile saline (0.85%, *w/v* NaCl) solution and homogenised in a Stomacher (BagMixer, interscience, Cantal, France) bag homogenizer, and subsequently serial dilutions were prepared. Tryptic soy agar (TSA) and oxytetracycline glucose yeast agar (OGYA) plates were used for total bacterial count and yeast count, respectively. The TSA plates were incubated at 37 °C for 24 h, and OGYA plates were at 30 °C for 72 h. Enumeration results of bacterial counts were expressed as log CFU/g cheese [60].

##### Sensory Assessment

Polyelectrolyte polymers-based edible hydrogel film-coated (PCLLa and CLLA) and Perline Mozzarella cheese substrate (control) samples were evaluated for sensory characteristics of their colour, appearance, flavour (odour, saltiness, bitterness, sourness) and overall acceptability [66]. We followed the protocols of the Ethics Committee of Gebze Technical University (protocol details: 09.09.2024–2024/16–03 date and session with protocol number of 16–02). The sensory assessment was carried out by seven (7) professional and expert volunteers.

#### 4.4.3. In Vitro Lactase Enzyme Release Behaviour Analysis

In vitro gastro-intestinal digestion of PCLLa, CLLA and Perline Mozzarella cheese substrate (control) samples was performed following the protocol [73]. The gastrointestinal model liquids, simulated salivary (SSF), gastric (SGF) and intestinal (SIF) [74] fluids were prepared to investigate the release of lactase enzyme at body temperature (37 °C). Salivary phase for mouth fluid model was carried out by mixing 4 g of the samples with 20 mL of SSF containing 100 U mL^−1^ of human salivary α-amylase and incubating the samples at 37 °C for 5 min in a shaking incubator (120 rpm). Next, for the gastric step, 20 mL of SGF (containing 4000 U mL^−1^ of porcine pepsin and 120 U mL^−1^ of porcine gastric lipase) was added, the pH was adjusted to 3 and the samples further incubated at 37 °C for 2 h in a shaking incubator (120 rpm). Finally, the intestinal step was performed with the addition of 100 mL of SIF (containing 1 mmol L^−1^ of pancreatin), adjusting the pH to 6.7 and incubating the samples at 37 °C for 2 h in a shaking incubator (120 rpm). At the end of the digestion, the samples were centrifuged (5 min, 10,000 rpm) and lactase activities at each step were determined. For this, 200 µL supernatant samples were placed into Eppendorf tubes (Hamburg, Germany), and 200 µL ONPG (*ortho*-Nitrophenyl-β-D-galactopyranoside) solution (Sigma Aldrich, Rockville, MD, USA) was added and incubated at 37 °C for 15 min. Subsequently, the reaction was stopped by adding 200 µL NaCO_3_. Absorbance values were measured using a UV-VIS spectrophotometer at 420 nm. A control sample containing phosphate buffer instead of cheese was also used. A standard curve was constructed using *o*-nitrophenol (0–20 µg mL^−1^) to calculate enzyme activity. One unit of enzyme activity (U mL^−1^ min^−1^) was defined as the amount of enzyme that releases 1 µg of *o*-nitrophenol in one minute.

#### 4.4.4. Statistical Analysis

All collected results were presented as mean ± standard deviation, with three parallel experimental replicates. One-way analysis of variance and independent samples *t*-test were performed for data assessment using SPSS 22.0 software (SPSS Inc., Chicago, IL, USA). *p* < 0.05 was considered as statistically significant.

## Figures and Tables

**Table 1:** Liposomal encapsulation of active compounds; their encapsulation method, zeta potential, particle size and encapsulation efficiency.

Active Compounds Encapsulated in Liposomes	Encapsulation Method	Zeta Potential (mV)	Particle Size (nm)	Encapsulation Efficiency of Liposomes (%)	References
No active compound encapsulated	Dehydration-rehydration	−12.5 ± 0.9	601 ± 76	No encapsulation	[26]
Apple peel polyphenols	Reverse phase evaporation and sonication	−38.39 ± 0.25	267 ± 1.04	86.52 ± 0.60	[34]
Curcumin	Thin film hydration and sonication	−6.34 ± 0.23	102.15 ± 2.17	92.4	[35]
Clove essential oil	Ethanol injection and sonication	−21.99 ± 0.67	217.27 ± 1.18	66.60 ± 2.19	[36]
Papain hydrolysate enzyme	Thin film hydration and sonication	~−47	285.33 ± 9.45	79.40 ± 0.059	[37]
Casein peptides	Thin film hydration and homogenization	~−10	86.13 ± 0.62	87.29 ± 0.82	[38]
Anthocyanins	Ethanol injection and ultrasonication	−42.7 ± 2.2	53.8 ± 1.8	91.1 ± 1.7	[39]
Tea polyphenols	Thin film hydration and ultrasonication	−56.11 ± 4.34	132.01 ± 1.053	92.24 ± 0.168	[40]
Vitamin C	Microfluidic method	−19.7 ± 3.15	101	79.86 ± 1.20	[41]
Gentamicin and Curcumin	Microfluidic method	NR	173.03 ± 0.75	95.06 ± 0.31 94.69 ± 0.08	[42]
Olive leaf extract	Supercritical fluids technique	NR	300–710	86.0	[43]
Rosemary extract	Mozafari method	−65.1	583.5	54.59	[44]
Lactase enzyme	Probe sonication	−39.2	175.6	>90	Present study

NR: Not reported.

**Table 2:** Effects of edible coating on the pH changes in cheese during storage at 4 °C.

Samples	pH				
	Day 0	Day 15	Day 30	Day 45	Day 60
Control	5.69 ± 0.08 ^Ab^	5.95 ± 0.02 ^Ba^	5.93 ± 0.03 ^Aa^	5.94 ± 0.01 ^Aa^	5.96 ± 0.01 ^Aa^
PCLLa	5.70 ± 0.01 ^Ad^	5.98 ± 0.01 ^Aa^	5.89 ± 0.01 ^Bb^	5.90 ± 0.04 ^Bb^	5.83 ± 0.03 ^Bc^
CLLA	5.70 ± 0.00 ^Ab^	5.84 ± 0.01 ^Ca^	5.67 ± 0.01 ^Cc^	5.61 ± 0.01 ^Cd^	5.48 ± 0.01 ^Ce^

Values are presented as mean ± standard deviation. Different uppercase letters within the same column indicate significant differences among samples, while different lowercase letters within the same row indicate significant differences among storage days, according to Duncan (*p* < 0.05).

**Table 3:** Effects of the edible hydrogel films achieved on dry matter (total solids) of the cheese substrate during the period of their storage in 4 °C environment.

Samples	Total Solids (%)				
	Day 0	Day 15	Day 30	Day 45	Day 60
Control	43.0 ± 0.00 ^Ab^	44.01 ± 0.53 ^Aa^	43.78 ± 0.1 ^Aa^	44.04 ± 0.01 ^Aa^	42.55 ± 0.00 ^Ac^
PCLLa	43.0 ± 0.00 ^Aa^	43.04 ± 0.34 ^Ba^	42.04 ± 0.32 ^Bb^	42.57 ± 0.83 ^Bab^	40.26 ± 0.29 ^Bc^
CLLA	43.0 ± 0.00 ^Aa^	40.66 ± 0.3 ^Cb^	38.83 ± 0.17 ^Cc^	38.97 ± 0.72 ^Cc^	42.60 ± 0.49 ^Aa^

Values are presented as mean ± standard deviation. Different uppercase letters within the same column indicate significant differences among samples, while different lowercase letters within the same row indicate significant differences among storage days, according to Duncan (*p* < 0.05).

**Table 4:** Effects of the edible hydrogel films achieved on colour change in the cheese substrate during the period of their storage in 4 °C environment.

Samples	Colour Parameters				
	L*				
	Day 0	Day 15	Day 30	Day 45	Day 60
Control	85.88 ± 1.16 ^Aab^	86.75 ± 1.21 ^Aa^	83.80 ± 0.64 ^Cc^	84.84 ± 0.39 ^Bbc^	83.64 ± 0.26 ^Bc^
PCLLa	87.98 ± 1.22 ^Aa^	86.03 ± 1.32 ^Ab^	85.68 ± 0.87 ^B^	84.46 ± 0.18 ^Bc^	82.81 ± 0.72 ^Bd^
CLLA	83.23 ± 1.84 ^Bc^	87.15 ± 0.64 ^Aab^	87.89 ± 0.47 ^Aa^	88.31 ± 0.71 ^Aa^	86.26 ± 0.79 ^Ab^
	a*				
	Day 0	Day 15	Day 30	Day 45	Day 60
Control	−2.24 ± 0.12 ^Aa^	−2.58 ± 0.14 ^ABbc^	−2.43 ± 0.12 ^Ab^	−2.49 ± 0.09 ^Ab^	−2.74 ± 0.11 ^Ac^
PCLLa	−2.1 ± 0.12 ^Aa^	−2.69 ± 0.07 ^Bb^	−2.86 ± 0.05 ^Bc^	−2.98 ± 0.06 ^Bc^	−2.96 ± 0.11 ^Ac^
CLLA	−2.03 ± 0.26 ^Aa^	−2.47 ± 0.06 ^Aa^	−2.44 ± 0.03 ^Aa^	−2.42 ± 0.11 ^Aa^	−2.52 ± 0.07 ^Aa^
	b*				
	Day 0	Day 15	Day 30	Day 45	Day 60
Control	8.55 ± 0.73 ^Ab^	10.45 ± 0.65 ^Aa^	10.92 ± 0.85 ^Aa^	10.23 ± 0.94 ^Aa^	10.52 ± 0.74 ^Ba^
PCLLa	8.65 ± 0.69 ^Ac^	10.44 ± 0.55 ^Ab^	10.43 ± 0.22 ^Ab^	11.14 ± 0.1 ^Aa^	11.04 ± 0.29 ^ABab^
CLLA	9.72 ± 0.8 ^Ac^	10.19 ± 0.37 ^Abc^	10.66 ± 0.52 ^Aab^	10.85 ± 0.65 ^Aab^	11.49 ± 0.39 ^Aa^

Values are presented as mean ± standard deviation. L*, a*, and b* parameters were analyzed separately; within each parameter, different uppercase letters within columns and lowercase letters within rows indicate significant differences among samples and storage days, respectively, according to Duncan (*p* < 0.05).

**Table 5:** Effects of the edible hydrogel films achieved on the total growth amount of bacteria and yeasts of the cheese substrate during the period of their storage in 4 °C environment.

Microbiological Analyses					
Sample	Total Bacteria (log CFU/g)				
	Day 0	Day 15	Day 30	Day 45	Day 60
Control	7.94 ± 0.04 ^Aa^	7.57 ± 0.08 ^Ab^	7.52 ± 0.09 ^Bb^	7.54 ± 0.02 ^Cb^	7.57 ± 0.02 ^Bb^
PCLLa	7.94 ± 0.04 ^Aa^	7.6 ± 0.01 ^Ad^	7.74 ± 0.01 ^Ac^	7.79 ± 0.02 ^Abc^	7.81 ± 0.05 ^Ab^
CLLA	7.94 ± 0.04 ^Aa^	7.23 ± 0.13 ^Bd^	7.48 ± 0.06 ^Bc^	7.67 ± 0.02 ^Bb^	7.84 ± 0.02 ^Aa^
Sample	Total Yeast (log CFU/g)				
	Day 0	Day 15	Day 30	Day 45	Day 60
Control	6.45 ± 0.2 ^Ab^	7.43 ± 0.04 ^Aa^	7.52 ± 0.02 ^Ba^	7.5 ± 0.09 ^ABa^	7.55 ± 0.05 ^Ba^
PCLLa	6.45 ± 0.2 ^Ac^	7.29 ± 0.06 ^Bb^	7.38 ± 0.02 ^Cb^	7.41 ± 0.04 ^Bb^	7.66 ± 0.03 ^Aa^
CLLA	6.45 ± 0.2 ^Ab^	7.55 ± 0.09 ^Aa^	7.6 ± 0.02 ^Aa^	7.54 ± 0.02 ^Aa^	7.66 ± 0.02 ^Aa^

Values are presented as mean ± standard deviation. Total Bacteria and Total Yeast were analyzed separately; within each dataset, different uppercase letters within columns and lowercase letters within rows indicate significant differences among samples and storage days, respectively, according to Duncan (*p* < 0.05).

**Table 6:** Sensory analyses of cheese samples during shelf life.

Sample	Days	Color	Appearance	Odor	Bitterness	Sourness	Saltiness	Overall Acceptability
Control	0	4.57 ± 0.53 ^Aab^	4.71 ± 0.49 ^Aa^	4.43 ± 0.53 ^Aa^	4.86 ± 0.38 ^Aa^	4.14 ± 0.90 ^Aa^	4.00 ± 0.58 ^Ba^	3.71 ± 0.95 ^Aa^
	15	5.00 ± 0.00 ^Aa^	4.86 ± 0.38 ^Aa^	4.29 ± 0.95 ^Aa^	5.00 ± 0.00 ^Aa^	4.29 ± 0.95 ^Aa^	4.00 ± 0.82 ^Aa^	4.43 ± 0.53 ^Aa^
	30	4.29 ± 0.76 ^Aab^	4.29 ± 0.76 ^Aab^	4.00 ± 0.58 ^Aa^	5.00 ± 0.00 ^Aa^	4.29 ± 0.95 ^Aa^	3.57 ± 0.98 ^Aa^	4.00 ± 0.58 ^Aa^
	45	3.86 ± 0.90 ^Bb^	3.86 ± 0.90 ^Bb^	4.43 ± 0.79 ^Aa^	5.00 ± 0.00 ^Aa^	4.29 ± 0.76 ^Aa^	4.14 ± 0.69 ^Aa^	4.14 ± 0.69 ^Aa^
	60	4.14 ± 0.90 ^ABb^	4.14 ± 0.90 ^Aab^	4.00 ± 1.00 ^Aa^	5.00 ± 0.00 ^Aa^	4.57 ± 0.79 ^Aa^	4.29 ± 0.76 ^Aa^	4.29 ± 0.49 ^Aa^
PCLLa	0	4.71 ± 0.49 ^Aa^	4.57 ± 0.79 ^Aa^	4.43 ± 0.79 ^Aa^	5.00 ± 0.00 ^Aa^	4.00 ± 0.82 ^Aa^	4.57 ± 0.53 ^ABa^	4.14 ± 0.38 ^Aa^
	15	4.71 ± 0.76 ^Aa^	4.57 ± 0.79 ^Aa^	4.29 ± 0.95 ^Aa^	5.00 ± 0.00 ^Aa^	4.57 ± 0.79 ^Aa^	4.00 ± 0.82 ^Aa^	4.43 ± 0.79 ^Aa^
	30	4.14 ± 0.69 ^Aab^	4.43 ± 0.53 ^Aa^	4.00 ± 0.82 ^Aa^	5.00 ± 0.00 ^Aa^	4.43 ± 0.79 ^Aa^	3.71 ± 1.11 ^Aa^	4.14 ± 0.69 ^Aa^
	45	4.43 ± 0.53 ^ABab^	4.43 ± 0.53 ^ABa^	4.43 ± 0.79 ^Aa^	5.00 ± 0.00 ^Aa^	4.14 ± 0.90 ^Aa^	4.14 ± 0.69 ^Aa^	3.86 ± 0.69 ^Aa^
	60	3.86 ± 0.90 ^Bb^	4.00 ± 0.82 ^Aa^	4.14 ± 0.90 ^Aa^	5.00 ± 0.00 ^Aa^	4.71 ± 0.49 ^Aa^	4.43 ± 0.79 ^Aa^	4.43 ± 0.79 ^Aa^
CLLA	0	4.43 ± 0.79 ^Aa^	4.71 ± 0.49 ^Aa^	4.71 ± 0.49 ^Aa^	5.00 ± 0.00 ^Aa^	4.14 ± 0.69 ^Aa^	4.71 ± 0.49 ^Aa^	4.29 ± 0.49 ^Aa^
	15	4.57 ± 0.79 ^Aa^	4.43 ± 0.79 ^Aa^	4.29 ± 0.76 ^Aa^	5.00 ± 0.00 ^Aa^	4.14 ± 1.07 ^Aa^	4.00 ± 0.58 ^Aa^	4.43 ± 0.53 ^Aa^
	30	4.86 ± 0.38 ^Aa^	4.57 ± 0.53 ^Aa^	4.29 ± 0.76 ^Aa^	5.00 ± 0.00 ^Aa^	4.14 ± 0.90 ^Aa^	3.86 ± 1.21 ^Aa^	4.14 ± 0.69 ^Aa^
	45	4.86 ± 0.38 ^Aa^	4.71 ± 0.49 ^Aa^	4.71 ± 0.49 ^Aa^	4.86 ± 0.38 ^Aa^	4.14 ± 0.90 ^Aa^	4.14 ± 0.69 ^Aa^	4.29 ± 0.76 ^Aa^
	60	4.86 ± 0.38 ^Aa^	4.86 ± 0.38 ^Aa^	4.14 ± 0.69 ^Aa^	5.00 ± 0.00 ^Aa^	4.57 ± 0.79 ^Aa^	4.14 ± 1.07 ^Aa^	4.29 ± 0.76 ^Aa^

Values are presented as mean ± standard deviation. For each sensory attribute, different uppercase letters within the same day indicate significant differences among samples, while different lowercase letters within the same sample indicate significant differences among storage days, according to Duncan (*p* < 0.05).

## Data Availability

The raw data supporting the conclusions of this article will be made available by the authors on request.

## Supplementary Materials

The following supporting information can be downloaded at https://www.mdpi.com/article/10.3390/gels12040343/s1, Figure S1: FTIR spectra comparing pure chitosan polymer with the chitosan film developed in this study.; Figure S2: FTIR spectra comparing pure alginate polymer with the alginate film developed in this study.
