# Supplementary material for: Enzyme-Loaded Liposomal Edible Hydrogel Films to Enhance Lactase Activity in Perline Mozzarella
Source: Gels. 2026 Apr 20;12(4):343. doi: 10.3390/gels12040343 (PMC13116243; doi:10.3390/gels12040343)
Supplement: Supplementary file 1 [file gels-12-00343-s001.zip › gels-4179563-supplementary.pdf]

Supplementary Data

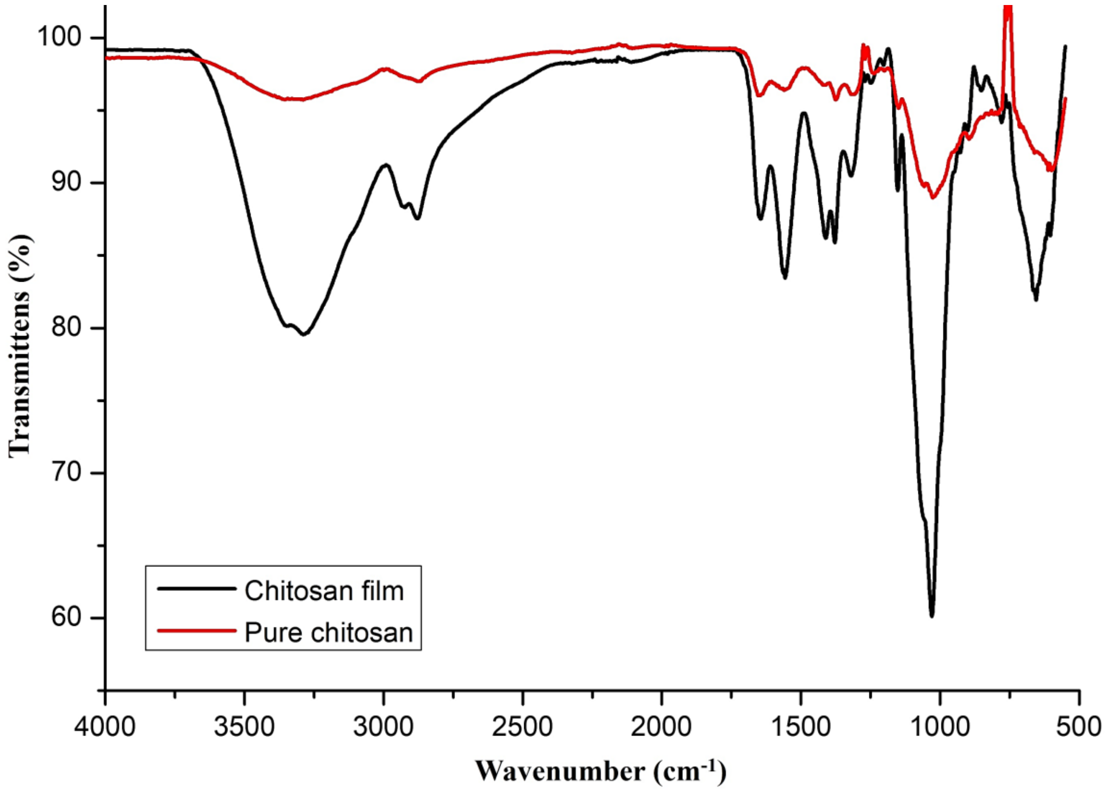

Figure S1: FTIR spectra comparing pure chitosan polymer with the chitosan film developed in this study.

The FTIR spectra of pure chitosan in powder form and chitosan films showed significant alterations as seen in Figure S1. The broad band between 3500 cm^{-1}–3300 cm^{-1} was –NH stretching vibrations in amino group and –OH group vibrations. They got broader and wider representing that the molecular interactions occurred while chitosan film production. The C-H streching vibrations between 3000 cm^{-1}–2850 cm^{-1} got more intense in film form. 1590 cm^{-1} was the stretching vibration band of amino group of chitosan indicating that the characteristic amino structure of chitosan was preserved. At 1642cm^{-1}: C=O stretching (amide I) and at 1652 cm^{-1}: amino group –NH bending vibrations and at 1410 cm^{-1}–1420 cm^{-1}–CH_2 bending vibrations are recorded. The asymmetric vibrations at around 1150 cm^{-1} and 885 cm^{-1} were –C–O–C bridges from the glucopyranose ring in chitosan matrix presented in fingerprint region. This sharp peak indicated that the polymeric film structure formed.

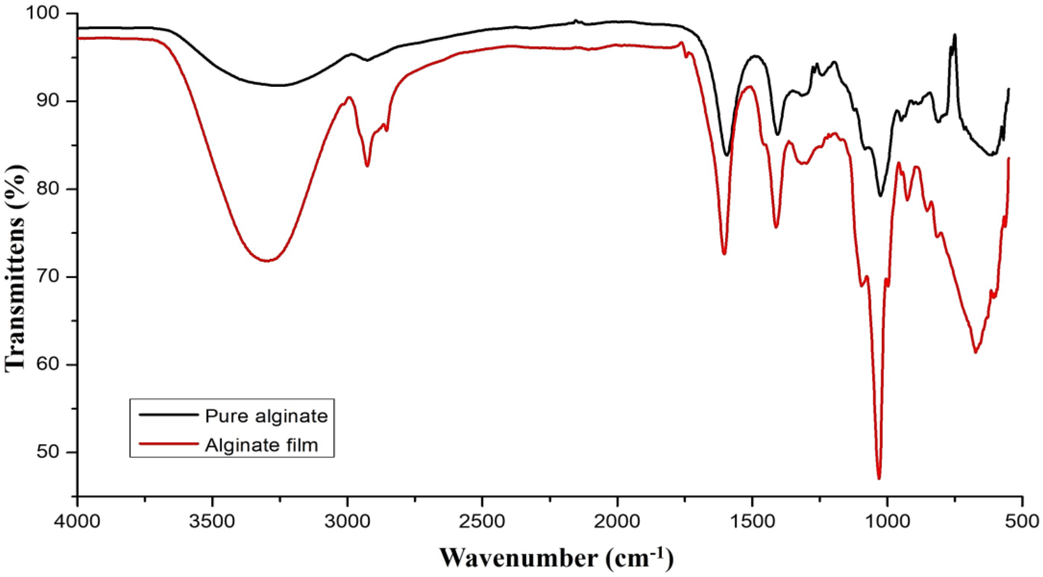

Figure S2: FTIR spectra comparing pure alginate polymer with the alginate film developed in this study.

In Figure S2, a strong broad band around 3200 cm^{-1}–3400 cm^{-1} was assigned to O–H stretching vibrations that indicates the formation of interactions between molecules; alginate chains come closer and form film structure. The other characteristic peaks of the alginate films were observed at 2927 cm^{-1} was associated with stretching vibration absorbance of C–H, these vibrations in the backbone got more intense via film formation. The peaks between 1600 cm^{-1}–1400 cm^{-1} were asymmetric and symmetric stretching vibrations of carboxylate ions (–COO) of alginate. They got more stabilized in the film form. 1090 cm^{-1}–1040 cm^{-1} represents absorption bands obtained in the range of 1090 cm^{-1} to 1040 cm^{-1} could be attributed to the stretching of –C=O and the hydroxyl group. 1030 cm^{-1}: The sharp peak that occurred at 1030 cm^{-1} indicated that the carboxylic groups of sodium alginate found in the main skeletal structure were preserved. The sharp peak occurred between 920 cm^{-1}–1100 cm^{-1} was C–O–C skeletal stretching of alginate in fingerprint region. 812 cm^{-1}–920 cm^{-1}: C–H bending in mannuronic units of alginate had wider and broader in the film forming structure.
